# Cell-free Production of the Extracellular Domain of the Nicotinic Acetylcholine Receptor

**Published:** 2009-04

**Authors:** E.N. Lyukmanova, G.S. Kopeina, M.A. Shulepko, Z.O. Shenkarev, A.S. Arseniev, D.A. Dolgikh, M.P. Kirpichnikov

**Affiliations:** 1 Shemyakin-Ovchinnikov Insitute of Bioorganic Chemistry, Russian Academy of Sciences, 16/10, Miklucho-Maklaya str., Moscow, Russia;; 2 Moscow State University, Leninskie Gory, GSP-1, Moscow, 119991

## 

The nicotinic acetylcholine receptor (nAChR) is a ligand-gated ion channel which is incorporated into the postsynaptic membrane of neurons [1]. NAChR is composed of five homologous subunits, whose transmembrane domains form an ion pore and the N-terminal domains contain the binding sites for the ligand (Fig.1). One of the most common subtypes of nAChR receptor in mammalian nervous systems is a homopentameric, α7 nAChR (α7nAChR), and several neurodegenerative disorders are associated with its dysfunction [2]. An effective system for the production of the individual subunits and other domains of nAChR is a prerequisite for studies focused on the receptor itself, and for the design of biomedical drugs to be used in the treatment of the disease. The tendency of these proteins to form insoluble aggregates in solution makes the development of such systems difficult [3].

**Fig. 1.**
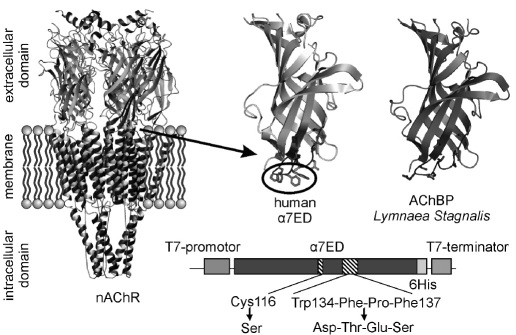
Model of the spatial organization of nAChR [1]. Comparison of a model of α7ED [6] with the spatial structure of AChBP subunit [4]. Scheme of mutations.

Recently, a research group from the USA published the x-ray structure of acetylcholine binding protein (AchBP) from Lymnaea stagnalis [4]. AChBP is a water-soluble protein composed of five identical subunits. This protein shares 25% sequence homology with the extracellular domain of α7nAChrR (α7ED), and it is capable of interacting with some of the nAChR ligands (for example, acetylcholine, α-conotixins, and α-neurotoxins) [4]. It has been shown that replacing the fragment of α7ED which is located between Cys128 and Cys142 (the so-called Cys-loop) with a homologous loop from AChBP increases the solubility of the domain [5].

During the past 10 years, cell-free systems, especially continuous-z exchange cell-free systems, have been successfully used in the production of recombinant proteins [7, 8]. These systems have some advantages over host-based gene expression systems: (i) the direct addition of special agents or co-factors into the reaction mixture can prevent the aggregation of the target protein, and (ii) the method permits the synthesis of selectively labeled proteins which can then be used in structural studies. 

The main objective of the present study was the development of an effective cell-free system for the production of human α7ED. For this purpose, from a full-length gene of α7nAChR (a generous gift from Prof. J. Lindstrom) two mutant α7ED genes were constructed, with substitutions that were intended to increase solubility. The first gene (α7ED/C116S/Cys-loop) coded for α7ED with two substitutions: (i) unpaired Cys116 was replaced by Ser [3], and (ii) Cys-loop of α7ED (Cys128-Cys142) was replaced by Cys-loop from AChBP [5]. An analysis of the model of α7ED [6] revealed that only a small fragment (Trp134-Phe-Pro-Phe137) of the Cys-loop is hydrophobic in nature and that it probably interacts with the membrane portion of the receptor (Fig.1). At the same time, the homologous region of AChBP contains only hydrophilic residues (Fig.1). Thus, the second mutant gene (α7ED/C116S/DTES) encodes α7ED with the replacement of this hydrophobic site with Asp-Thr-Glu-Ser from AChBP and the substitution of Cys116Ser.

Mutant genes, with an additional sequence coding for a His 6-tag on the C-terminus, were cloned into the pET22b(+) vector. In both cases, the majority of the synthesized protein was in the form of insoluble aggregates, and only a small percentage remained soluble (Fig.2, lanes 2-4). The most likely cause for the observed aggregation is the incorrect formation of disulfide bonds and/or the tendency of α7ED toward spontaneous pentamerization when in solution [9]. The addition of reduced (GSH) and oxidized (GSSG) glutathiones, in concentrations of 0.1 mM and 0.5 mM, respectively, to the translation solution increased the yield of soluble proteins by up to 30% (Fig.2, lanes 5-7). The presence in the translation solution of a low-molecular-weight nAChR agonist (carbamylcholine, CCh) yielded similar results (Fig.2, lanes 8-10). The use of a soft, non-ionic detergent (Brij-35) at a concentration of 0.5% to prevent spontaneous pentamerization, together with GSH and GSSG, caused a significant decrease in the fraction of insoluble protein (Fig.2, lanes 11-13). It should be noted that both chimeras of α7ED displayed identical properties during the process of synthesis and during subsequent manipulations, so the experiment proceeded using only α7ED/C116S/DTES.

**Fig. 2.**
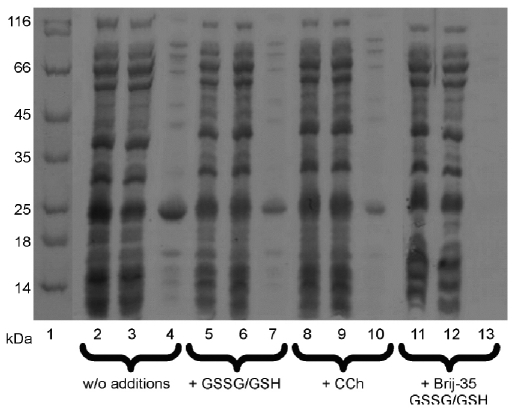
Electrophoresis analysis of the cell-free synthesis of α7ED/C116S/DTES. 1- markers of molecular weights; 2, 5, 8, 11 – total protein fraction of translation mixture; 3, 6, 9, 12 – soluble protein fraction of translation mixture; 4, 7, 10, 13 - insoluble protein fraction of translation mixture.

Purification of the recombinant proteins using metal-affinity chromatography caused the proteins to precipitate. Thus, the conditions for the refolding of α7ED/C116S/DTES from a precipitate of the translation mixture were determined. Several different approaches (for example, 8M urea, 6M guanidine hydrochloride and 1% sodium laurylsarcosine, LS) for initial precipitate dissolution were tried. The highest efficiency was achieved using a mixture of 3M urea and 1% LS in the presence of DTT. The refolding of α7ED/C116S/DTES was carried out on metal-affinity resin by washing with GSSG/GSH in a descending concentration gradient of both LS and Urea. However, the protein that was obtained turned out to be highly unstable in solution. The alternative protocol involved the replacement of 1% LS with 0.1% β-dodecylmaltoside (DDM) or 0.1% dodecylphosphocholine (DPC). 

As a result, highly stable (more than 1 month at +4°С) protein preparations, with a final yield of 1 mg per ml of the translation mixture, were obtained. The samples of α7ED/C116S/DTES in DDM and DPC solution were analyzed using size-exclusion chromatography (Fig.3). In the case of DDM, recombinant α7ED was found in the form of large soluble aggregates (Fig.3). Use of DPC yielded a much more homogenous (>90%) sample with an average particle diameter of 7mm, which corresponds to a size of α7ED monomer (5 nm) associated with DPC micelle (4 nm). An analysis of the secondary structure of α7ED/C116S/DTES in DPC solution using CD spectroscopy revealed a prevalence of the β-structure, which was in keeping with our expectations (Fig.3). The ability of α7ED/C116S/DTES in DPC solution to interact with nAChR antagonists was studied using NMR spectroscopy with an 15N-labeled long-chain neurotoxin NTII/I obtained as in [10]. The NTII/I sample in DPC was titrated by α7ED/C116S/DTES, and an attenuation of the toxin signals in 1D 15N-HSQC spectra was observed (Fig. 4). An analysis of the attenuation curve revealed that one molecule of the domain cooperatively binds two molecules of the toxin (Hill coefficient approximately 1.8) and an apparent dissociation constant of approximately 2 mkM. 

**Fig. 3.**
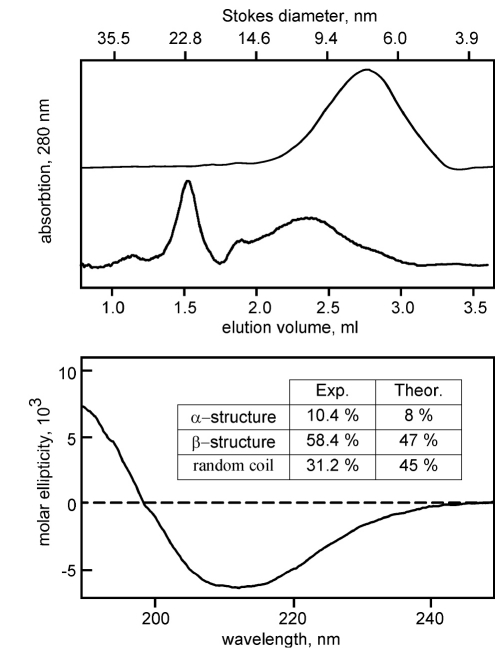
Analysis of α7ED/C116S/DTES in DDM and DPC solution using size-exclusion chromatography on Superdex-200 (GE Healthcare). CD spectrum of α7ED/C116S/DTES in DPC solution.

**Fig. 4.**
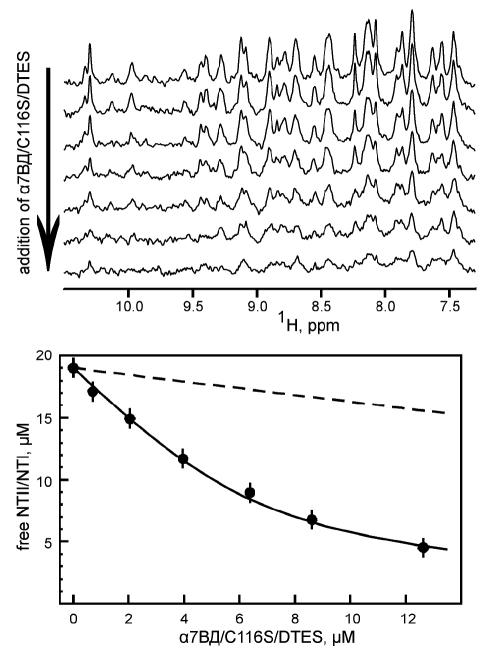
1D 15N-HSQC NMR spectrum of NTII/I toxin in the presence of DPC micelles and following an increase in α7ВД/C116S/DTES concentrations. The obtained isotherm binding and dilution curves (dashed line) are shown.

In summary, we developed a new cell-free system for the production of the active extracellular domain of α7nAChR. The addition of DPC to the protein sample stabilizes the domain in solution, preserves the secondary structure, and doesn't prevent the binding of antagonists. The development of this system creates new possibilities for future structural-functional studies of nAChR/ligand interactions. 

## Acknowledgements

This work was financially supported by the Russian Academy of Sciences (The program "Cell and Molecular Biology"), Russian Foundation for Basic Research, and by a grant from the President of the Russian Federation "young scientist" MK-6386.2008.4.
